# A Computational Solution to Bolster Epigenetic Clock Reliability for Clinical Trials and Longitudinal Tracking

**DOI:** 10.1093/geroni/igab046.015

**Published:** 2021-12-17

**Authors:** Albert Higgins-Chen, Kyra Thrush, Tina Hu-Seliger, Yunzhang Wang, Sara Hagg, Morgan Levine

**Affiliations:** 1 Yale University, New Haven, Connecticut, United States; 2 Elysium Health, New York, New York, United States; 3 Karolinska Institutet, Stockholm, Stockholms Lan, Sweden

## Abstract

Epigenetic clocks are widely used aging biomarkers, but they are calculated from methylation data for individual CpGs that can be surprisingly unreliable. We report that technical noise causes six major epigenetic clocks to deviate by 3 to 9 years between replicates. We present a novel computational solution: we perform principal component analysis followed by biological age prediction using principal components, extracting shared age-related changes across CpGs while ignoring noise from individual CpGs. Our novel principal-component versions of six clocks show agreement between most technical replicates within 1 year, and increased stability in short- and long-term longitudinal studies. This requires only one additional step compared to traditional clocks, does not require prior knowledge of CpG reliabilities, and can improve the reliability of any existing or future epigenetic biomarker. The extremely high reliability of principal component epigenetic clocks makes them particularly useful for personalized medicine and clinical trials evaluating novel aging interventions.

